# Geographical Variation in Social Determinants of Female Breast Cancer Mortality Across US Counties

**DOI:** 10.1001/jamanetworkopen.2023.33618

**Published:** 2023-09-14

**Authors:** Taylor Anderson, Dan Herrera, Franchesca Mireku, Kai Barner, Abigail Kokkinakis, Ha Dao, Amanda Webber, Alexandra Diaz Merida, Travis Gallo, Mariaelena Pierobon

**Affiliations:** 1 Department of Geography and Geoinformation Science, George Mason University, Fairfax, Virginia; 2 Department of Environmental Science and Technology, University of Maryland, College Park; 3 Department of Environmental Science and Policy, George Mason University, Fairfax, Virginia; 4 Department of Statistics, George Mason University, Fairfax, Virginia; 5 Department of Global and Community Health, George Mason University, Fairfax, Virginia; 6 School of Systems Biology, Center for Applied Proteomics and Molecular Medicine, George Mason University, Manassas, Virginia

## Abstract

**Importance:**

Breast cancer mortality is complex and traditional approaches that seek to identify determinants of mortality assume that their effects on mortality are stationary across geographic space and scales.

**Objective:**

To identify geographic variation in the associations of population demographics, environmental, lifestyle, and health care access with breast cancer mortality at the US county-level.

**Design, Setting, and Participants:**

This geospatial cross-sectional study used data from the Surveillance, Epidemiology, and End Results (SEER) database on adult female patients with breast cancer. Statistical and spatial analysis was completed using adjusted mortality rates from 2015 to 2019 for 2176 counties in the US. Data were analyzed July 2022.

**Exposures:**

County-level population demographics, environmental, lifestyle, and health care access variables were obtained from open data sources.

**Main Outcomes and Measures:**

Model coefficients describing the association between 18 variables and age-adjusted breast cancer mortality rate. Compared with a multivariable linear regression (OLS), multiscale geographically weighted regression (MGWR) relaxed the assumption of spatial stationarity and allowed for the magnitude, direction, and significance of coefficients to change across geographic space.

**Results:**

Both OLS and MGWR models agreed that county-level age-adjusted breast cancer mortality rates were significantly positively associated with obesity (OLS: β, 1.21; 95% CI, 0.88 to 1.54; mean [SD] MGWR: β, 0.72 [0.02]) and negatively associated with proportion of adults screened via mammograms (OLS: β, −1.27; 95% CI, −1.70 to −0.84; mean [SD] MGWR: β, −1.07 [0.16]). Furthermore, the MGWR model revealed that these 2 determinants were associated with a stationary effect on mortality across the US. However, the MGWR model provided important insights on other county-level factors differentially associated with breast cancer mortality across the US. Both models agreed that smoking (OLS: β, −0.65; 95% CI, −0.98 to −0.32; mean [SD] MGWR: β, −0.75 [0.92]), food environment index (OLS: β, −1.35; 95% CI, −1.72 to −0.98; mean [SD] MGWR: β, −1.69 [0.70]), exercise opportunities (OLS: β, −0.56; 95% CI, −0.91 to −0.21; mean [SD] MGWR: β, −0.59 [0.81]), racial segregation (OLS: β, −0.60; 95% CI, −0.89 to −0.31; mean [SD] MGWR: β, −0.47 [0.41]), mental health care physician ratio (OLS: β, −0.93; 95% CI, −1.44 to −0.42; mean [SD] MGWR: β, −0.48 [0.92]), and primary care physician ratio (OLS: β, −1.46; 95% CI, −2.13 to −0.79; mean [SD] MGWR: β, −1.06 [0.57]) were negatively associated with breast cancer mortality, and that light pollution was positively associated (OLS: β, 0.48; 95% CI, 0.24 to 0.72; mean [SD] MGWR: β, 0.27 [0.04]). But in the MGWR model, the magnitude of effect sizes and significance varied across geographical regions. Inversely, the OLS model found that disability was not a significant variable for breast cancer mortality, yet the MGWR model found that it was significantly positively associated in some geographical locations.

**Conclusions and Relevance:**

This cross-sectional study found that not all social determinants associated with breast cancer mortality are spatially stationary and provides spatially explicit insights for public health practitioners to guide geographically targeted interventions.

## Key Points

**Question:**

How do associations between county-level age-adjusted breast cancer mortality and population demographic, environmental, lifestyle, and health care access characteristics vary geographically in the US?

**Findings:**

This cross-sectional study of 2176 US counties found that the statistically significant positive association between obesity and breast cancer mortality was consistent across all counties in the US, but that access to factors in the built environment to support a healthy lifestyle had varying associations with mortality based on the county in which an individual lives.

**Meaning:**

These results suggest that breast cancer mortality in the US can be affected by where individuals live, and that more comprehensive and geographically targeted interventions may lead to healthier communities.

## Teaser

This cross-sectional study examines US county-level data for breast cancer mortality to detail regional variations in the association between mortality and demographic, lifestyle, and clinical variables.

## Introduction

Breast cancer is the leading cause of cancer-related deaths among women in the US.^1^ Biological and behavioral determinants of breast cancer mortality are generally known and have guided successful interventions and prevention programs that target individuals at risk.^2,3,4^ However, due to the complex interrelation between individual and contextual determinants, geographic disparities in breast cancer mortality remain difficult to address.^5,6^

While traditional regression approaches, commonly used in urban health research, have been useful in identifying determinants of breast cancer mortality, they are limited in that they assume spatial stationarity, meaning that one measure is used to describe the association between the independent and response variable for the entire area under study. Toward addressing this assumption, spatial approaches such as geographically weighted regression (GWR)^7^ and geographical random forest (GRF)^8^ compute local associations or the relative importance of variables and breast cancer mortality for each geographic unit within the study area.^9,10,11^ However, these approaches disregard the possibility that variables affecting breast cancer likely manifest at different spatial scales. For example, on a smaller scale, neighborhoods may have varying degrees of access to exercise opportunities. On a larger scale, states may fund different programs that support remission care for uninsured individuals.

**Figure 1.**
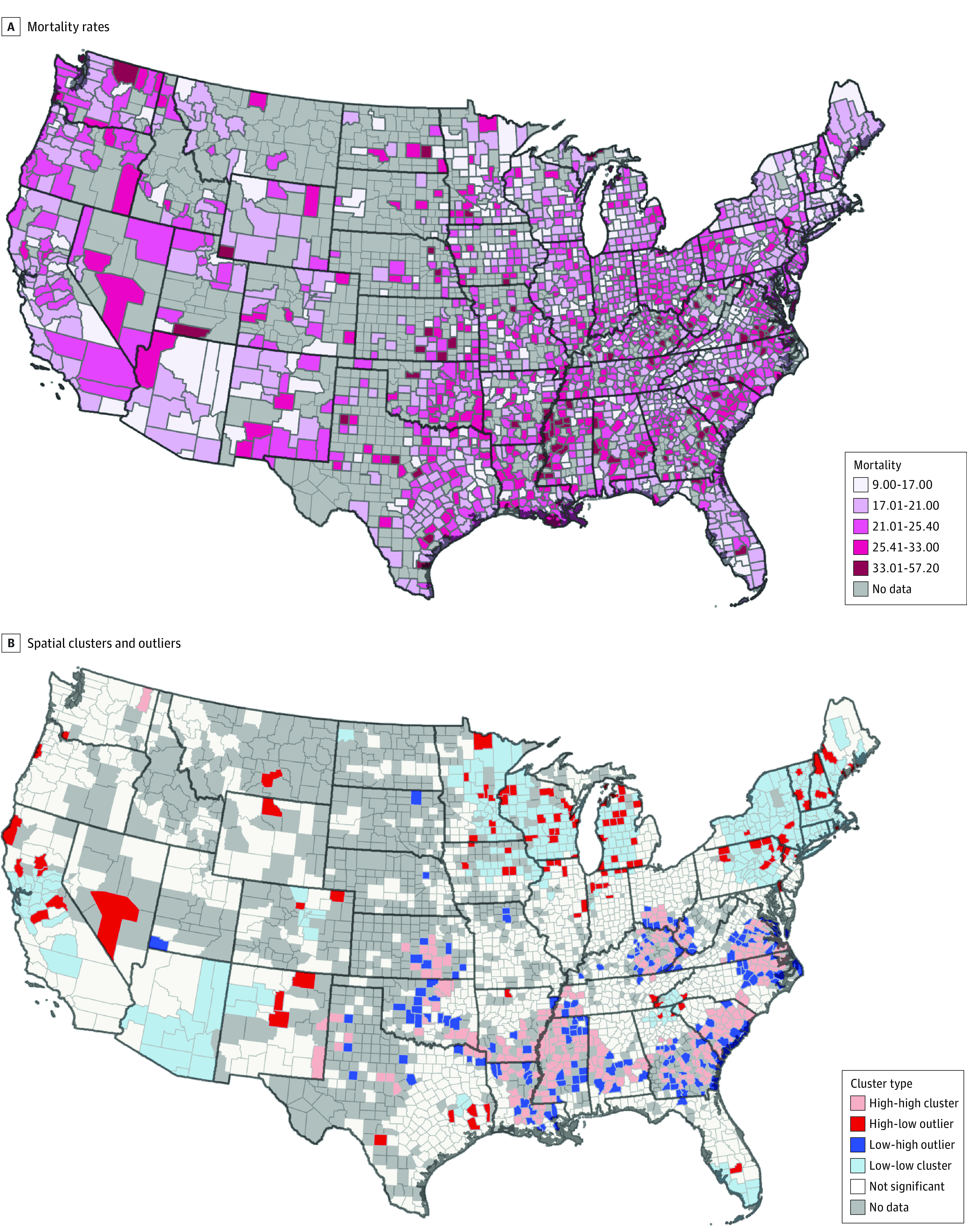
Female Breast Cancer Mortality Rates From 2015 to 2019 and Spatial Clusters and Outliers Mortality rate is defined as the number of deaths per 100 000 women per year. Cluster type refers to features of counties from Local Moran I statistics surrounded by counties with alike features, and outliers as counties surrounded by counties with different features—eg, high-high clusters indicate counties with high breast cancer mortality rates surrounded by counties that also had high rates, and high-low outliers indicate counties with high breast cancer mortality surrounded by counties with low rates.

The spatial heterogeneity of breast cancer mortality across the US (Figure 1A) presents an opportunity to explore the contextual and environmental variables that might give rise to such spatial disparities and the potential for nonstationarity in these data across space and scales. One such approach, multiscale geographically weighted regression (MGWR), is an extension of GWR that allows for the association between determinants and breast cancer mortality to vary both across geographic space and at different scales.^12^ Therefore, the objective of this geospatial cross-sectional study is to identify county-level social determinants of health including population demographics, environment, lifestyle, health care access, and pollutant variables using MGWR to address both spatial heterogeneity and the effects of scale on breast cancer mortality. We focus primarily on age-adjusted female breast cancer mortality as our dependent variable, which normalizes county mortality rates based on age characteristics of the corresponding county using 2000 US census data.^13,14^ The goal of this study is to enable location-specific interventions that can be addressed at various levels of public health.

## Methods

### Source of Data

#### Outcome

For each US county, excluding Alaska and Hawaii, age-adjusted female breast cancer mortality rates from 2015 to 2019 were retrieved from the Surveillance, Epidemiology, and End Results (SEER) database version 8.4.0.1^15^ in June of 2022 (Figure 1A). Female breast cancer mortality rate is defined as the number of deaths per 100 000 women per year and age-adjusted rates are standardized to the 2000 US population.^14^ Approximately one-third of the 3108 counties that make up the contiguous US (932 counties for results on age-adjusted rates) had no reported data, which is standard practice for counties reporting less than 10 deaths from 2015 to 2019. Thus, these counties were excluded from the analysis. Since all data were publicly available and deidentified, neither informed consent nor institutional review board approval was required. This study followed the Strengthening the Reporting of Observational Studies in Epidemiology (STROBE) reporting guideline for cross-sectional studies.

#### Independent Variables

County-level data were retrieved for 57 social determinants selected a priori for their known association with breast cancer incidence and mortality (eTable 1 in Supplement 1). Independent variables were collected from the Social Vulnerability Index,^16^ County Health Rankings & Roadmaps,^17^ OpenStreetMap,^18^ raw points of interest data from SafeGraph, NASA Black Marble,^19^ the US National Land Cover Data set,^20,21^ and ClinicalTrials.gov (eAppendix in Supplement 1).^22^ Variables were subclassified into 5 main categories: access to health care (7 variables), sociodemographics of the population (24 variables), lifestyle (5 variables), physical environment (15 variables), and pollutant (6 variables).

Of the 57 total variables, 24 variables were removed due to collinearity (*r* > 0.6 or variance inflation factor above 3.0) (eTable 1 and eFigure 1 in Supplement 1).^23^ The remaining 33 variables were evaluated using a leaps algorithm^24^ in R version 2.2.1 (R Project for Statistical Computing) to determine the best subset of variables. When using age-adjusted female breast cancer mortality, the final variable set contained 18 variables (Table 1 and Table 2). We note that none of the variables from the pollutant category were selected in the final model due to poor predictive capability. Some variables were log transformed to improve model convergence. All variables were scaled to have a mean of zero with an SD of 1.

**Table 1.** Multivariable Linear Regression Results Using Age-Adjusted Female Breast Cancer Mortality Rates as Dependent Variable

County variable (N = 2174)^a^	Standardized β	SE (95% CI)	*P* value
Intercept	32.67	0.23 (32.23 to 33.11)	<.001
Lifestyle			
Smoking (% adults)	−0.65	0.17 (−0.98 to −0.32)	<.001
Obesity (% adults)	1.21	0.17 (0.88 to 1.54)	<.001
Food environment index	−1.35	0.19 (−1.72 to −0.98)	<.001
Long commute (% workers)	0.08	0.13 (−0.17 to 0.33)	.56
Exercise opportunities (% population)	−0.56	0.18 (−0.91 to −0.21)	.002
Population demographics			
Unemployment (% population aged ≥16 y)	−0.20	0.16 (−0.51 to 0.11)	.22
Segregation (total population:White ratio)	−0.60	0.15 (−0.89 to −0.31)	<.001
Disability (% population)	0.18	0.17 (−0.15 to 0.51)	.28
Income inequality (ratio 80th:20th percentile)	−0.15	0.15 (−0.44 to 0.14)	.31
Access to health care			
Uninsured (% population)	−0.32	0.15 (−0.61 to −0.03)	.03
Mammograms (% adults screened)	−1.27	0.22 (−1.70 to −0.84)	<.001
Mental health care physicians (ratio to total population)	−0.93	0.26 (−1.44 to −0.42)	<.001
Primary care physicians (ratio to total population)	−1.46	0.34 (−2.13 to −0.79)	<.001
Hospital access (No. per capita)	−0.26	0.15 (−0.55 to 0.03)	.09
Environment			
Mean radiance (Watts × cm^−2^ × sr^−1^)	0.48	0.12 (0.24 to 0.72)	<.001
Transit access (No. stops per capita)	−0.43	0.13 (−0.68 to −0.18)	.001
Proportion of natural land per county	0.02	0.14 (−0.25 to 0.29)	.91
Grocery stores (No. per capita)	0.52	0.19 (0.15 to 0.89)	.006

^a^ Basis for comparison between states included in parentheses.

**Table 2.** Multiscale Geographically Weighted Regression Results Using Age-Adjusted Female Breast Cancer Mortality Rates as the Dependent Variable

County variable (N = 2174)^a^	Mean standardized (SD) β estimate	Range	Counties with significant result, %^b^	Bandwidth
Intercept	22.76 (1.05)	19.19 to 26.78	100	89
Lifestyle				
Smoking (% adults)	−0.75 (0.92)	−2.99 to 1.50	16.3	259
Obesity (% adults)	0.72 (0.02)	0.67 to 0.75	100	2179
Food environment index	−1.69 (0.70)	−2.85 to 0.36	80.3	389
Long commute (% workers)	0.20 (0.04)	0.06 to 0.23	0	2179
Exercise opportunities (% population)	−0.59 (0.81)	−3.46 to 1.05	13.5	280
Population demographics				
Unemployment (% population aged ≥16 y)	−0.13 (0.06)	−0.31 to −0.10	0	2179
Segregation (total population:White ratio)	−0.47 (0.41)	−1.81 to 0.02	22.6	851
Disability (% population)	0.40 (0.17)	0.08 to 0.61	45.0	1672
Income inequality (80th:20th percentile ratio)	−0.30 (0.01)	−0.34 to −0.27	0	2179
Access to health care				
Uninsured (% population)	−0.17 (0.02)	−0.23 to −0.12	0	2179
Mammograms (% adults screened)	−1.07 (0.16)	−1.29 to −0.74	100	2059
Mental health care physicians (ratio to population)	−0.48 (0.92)	−2.40 to 2.21	14.0	418
Primary care physicians (ratio to population)	−1.06 (0.57)	−2.07 to −0.01	40.6	925
Hospital access (No. per capita)	−0.14 (0.03)	−0.18 to −0.04	0	2179
Environment				
Mean radiance (Watts × cm^−2^ × sr^−1^)	0.27 (0.04)	0.22 to 0.37	42.4	2179
Transit access (No. stops per capita)	−0.31 (0.02)	−0.34 to −0.24	83.0	2179
Proportion of natural land per county	0.10 (0.01)	0.08 to 0.11	0	2179
Grocery store access (No. per capita)	0.33 (0.05)	0.26 to 0.46	0	2179

^a^ Basis for comparison between states included in parentheses.

^b^ Threshold for significance *P* < .05.

### Statistical Analysis

To better visualize the spatial patterns of breast cancer mortality across the US, a cluster and outlier analysis of the age-adjusted breast cancer mortality rates were computed using a Local Moran I approach.^25^ Next, excluding counties with missing data, a linear regression model (OLS) was fit to the county-level data in which all variables were regressed against age-adjusted female breast cancer mortality. Linear models assume that a variable’s magnitude of effect is constant across the sample space.

To assess whether the effects of our independent variables vary geographically across the US, an MGWR model was also computed using the same variable sets. Unlike a linear model, MGWR allows the strength and direction of effect to vary across the sample space—potentially revealing county-specific variation in trends.^12^ Formally, MGWR computes a local regression model for every county (*i*) in the data set by borrowing data from other surrounding counties (*j*) that fall within county *i*’s neighborhood. The number of nearest neighbors from which data will be borrowed (that comprise *j*) is referred to as the bandwidth. MGWR recognizes that not all relationships occur at the same spatial scale. Thus, the bandwidth size varies for each variable, based on an optimization algorithm.

The MGWR model is expressed as:



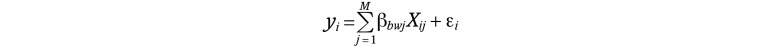



where *β_bwj_* is the estimation of the coefficient for county *i* and *bwj* is the optimal bandwidth size. The resulting bandwidths provide important information on the scale at which certain processes occur, thus indicating spatial nonstationarity. Smaller bandwidths indicate more local variation. Whereas larger values indicate a more global response similar to OLS. All statistical and spatial analysis were computed in ArcGIS Pro version 3.1.0 (Esri). Statistical significance was determined by 95% CIs. See eMethods in Supplement 1 for additional theoretical and technical details of the analyses.

## Results

The Local Moran I analysis identified spatial clusters and outliers of counties based on their age-adjusted breast cancer mortality rates (Figure 1B). A belt of counties with high breast cancer mortality rates (high-high cluster) was found to stretch from Kansas through Oklahoma east to Arkansas, Louisiana, Mississippi, Alabama, and Georgia and then up through South and North Carolina to Virginia. Another high-high cluster was observed along the borders of Kentucky, West Virginia, and Ohio. In contrast, clusters of counties with low breast cancer mortality rates (low-low cluster) were observed in California, Arizona, much of the Northeast, and parts of the Midwest. The map also highlights counties that have statistically high or low breast cancer mortality rates relative to their spatial neighbors (low-high outlier, high-low outlier). For example, Buffalo County, New York, has a much higher breast cancer mortality rate than the surrounding counties. In another example, Madison County, Tennessee, has a much lower breast cancer mortality rate than the surrounding counties.

We attempt to explain these spatial patterns of breast cancer mortality by comparing the coefficient of determination between risk factors and mortality rates using a conventional linear regression and the MGWR model. The MGWR was better at explaining the association between independent variables and breast cancer mortality rates across the US for adjusted mortality rates. For example, the linear model for age-adjusted female breast cancer mortality rates yielded an adjusted *R^2^* = 0.17 compared with an adjusted *R^2^* = 0.28 for the MGWR model (Tables 1 and 2, respectively).

**Figure 2.**
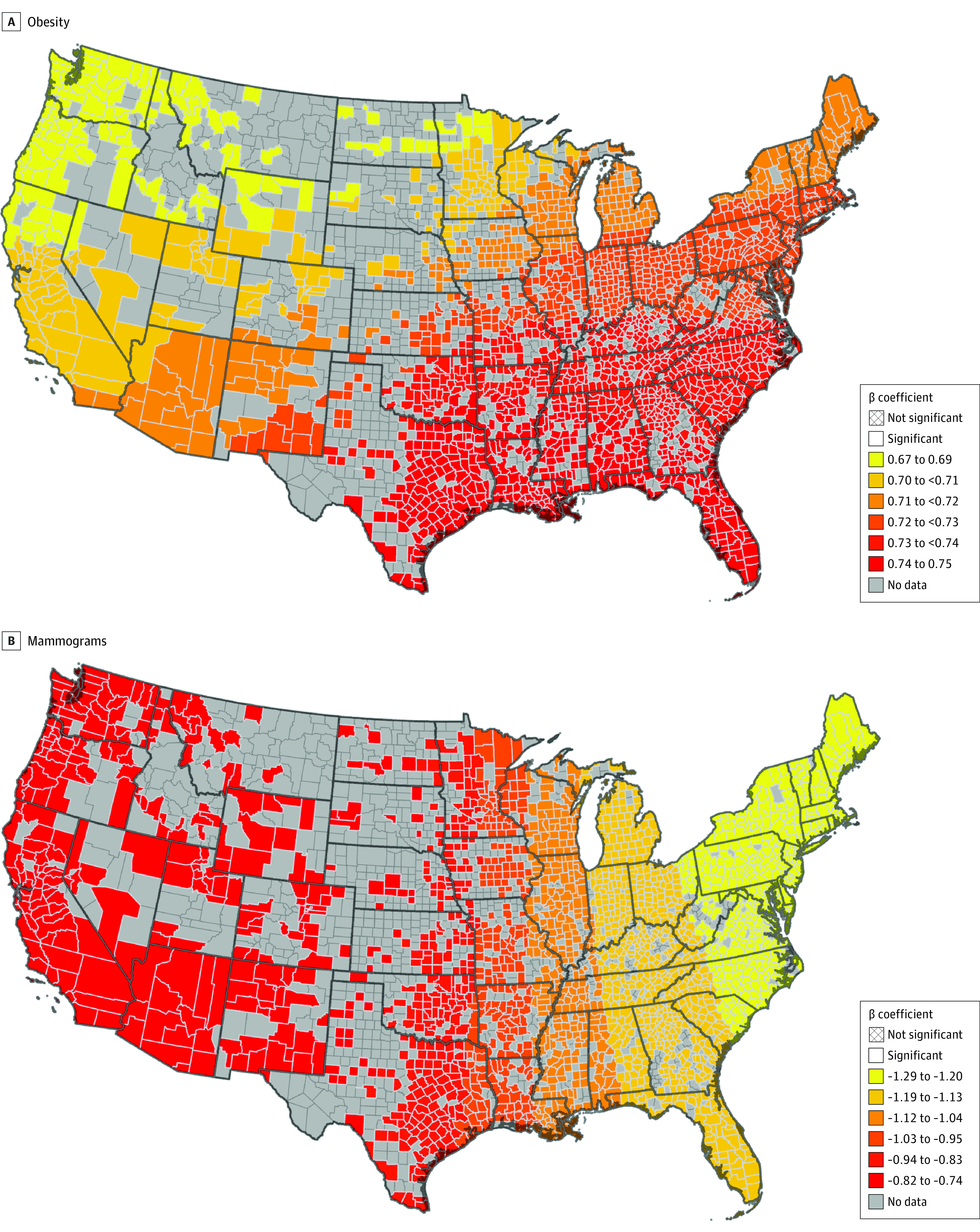
Multiscale Geographically Weighted Regression for the Association Between Age-Adjusted Female Breast Cancer Mortality and Spatially Stationary Variables Panel A, obesity and breast cancer mortality are positively associated; the association is spatially stationary across the US, although the effect size of the association is greater in the South. Panel B, mammogram testing and breast cancer mortality are negatively associated; the association is spatially stationary across the US, although the effect size is observed in the East.

A positive, statistically significant association between obesity and breast cancer mortality was observed in both the OLS (β, 1.21; 95% CI, 0.88 to 1.54; *P* < .001) and the MGWR (mean [SD] β, 0.72 [0.02]). Similarly, a negative and statistically significant association between the proportion of adults screened with mammograms and breast cancer mortality was observed in the OLS (β, −1.27; 95% CI, −1.70 to −0.84; *P* < .001) and the MGWR (mean [SD] β, −1.07 [0.16]). Furthermore, given that there are only small changes in the coefficients for obesity (Figure 2A) and proportion of adults screened for mammograms (Figure 2B), the MGWR results indicate that that the effects of these variables on mortality are spatially stationary.

The OLS and MGWR model agreed that in general breast cancer mortality was significantly negatively associated with smoking (OLS: β, −0.65; 95% CI, −0.98 to −0.32; *P* < .001; mean [SD] MGWR β, −0.75 [0.92]), food environment index (OLS: β, −1.35; 95% CI, −1.72 to −0.98]; *P* < .001; mean [SD] MGWR: β, −1.69 [0.70]), exercise opportunities (OLS: β, −0.56; 95% CI, −0.91 to −0.21; *P* = .002; mean [SD] MGWR: β, −0.59 [0.81]), segregation (OLS: β, −0.60; 95% CI, −0.89 to −0.31; *P* < .001; mean [SD] MGWR: β, −0.47 [0.41]), mental health care physician ratio (OLS: β, −0.93; 95% CI, −1.44 to −0.42; *P* < .001; mean [SD] MGWR: β, −0.48 [0.92]), and primary care physician ratio (OLS: β, −1.46; 95% CI, −2.13 to −0.79; *P* < .001; mean [SD] MGWR: β, −1.06 [0.57]), while positively associated with light pollution (mean radiance) (OLS: β, 0.48; 95% CI, 0.24 to 0.72; *P* < .001; mean [SD] MGWR: β, 0.27 [0.04]) (Tables 1 and 2).

**Figure 3.**
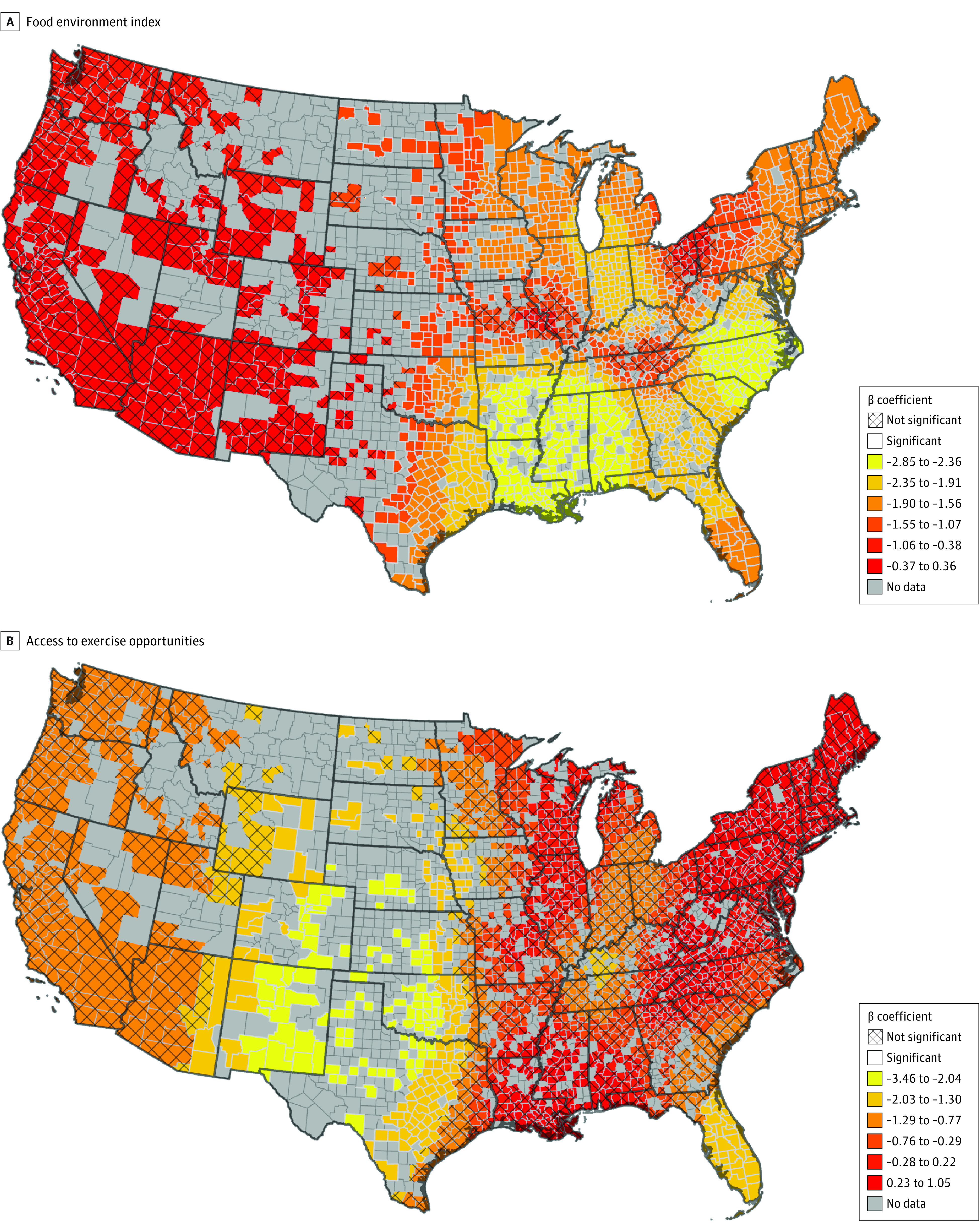
Multiscale Geographically Weighted Regression for the Association Between Age-Adjusted Female Breast Cancer Mortality and Spatially Nonstationary Variables Panel A, the association between food environment index and breast cancer mortality was spatially nonstationary, with the largest negative effect sizes in Louisiana, Arkansas, Alabama, North and South Carolina, and Virginia. B, the association between food environment index and breast cancer mortality is spatially nonstationary, with the largest negative effect sizes in central US and Florida.

However, while the OLS found that these variables are significant factors associated with breast cancer mortality overall, MGWR showed that they are only significant in some geographical locations. For example, where obesity and mammogram testing have a significant association with mortality in 100% of US counties, smoking had a significant effect in only 16.3%, food environment index in 80.3%, segregation in 22.6%, mental health care physician ratio in 14.0%, primary care physician ratio in 40.6%, and light pollution in 42.4%. Furthermore, the MGWR revealed that the magnitude of effect size of these variables varied from county to county, as demonstrated by the larger standard deviation of the beta coefficients and the smaller bandwidth sizes for these variables (Table 2). Thus, the association between these variables and breast cancer mortality can be considered spatially nonstationary with effects that vary regionally in scale. For example, the food environment index was not significantly associated with breast cancer mortality in the western US (Figure 3A). Yet, in most of the southern and eastern US, the food environment index was positively associated with breast cancer mortality with coefficients ranging from −1.55 to −2.85. This association had the largest effect sizes (ranging from β = −2.36 to β = −2.85) in Louisiana, Mississippi, Arkansas, and Alabama as well as North Carolina and parts of South Carolina and Virginia. Additionally, where access to exercise opportunities and breast cancer mortality was not significant for most of the US, a positive association with coefficients ranging from −1.30 to −3.46 was found in central US and Florida (Figure 3B).

Finally, where OLS estimated that disability was not significant, the MGWR estimated that it was significant in 45% of counties and that on average it was positively associated with breast cancer mortality (mean [SD] MGWR β, 0.4 [0.17]). In contrast, where OLS found a negative association between the uninsured and breast cancer mortality (β, −0.32; 95% CI, −0.61 to −0.03; *P* = .03), the MGWR found that the coefficients for this variable were not statistically significant for any county in the US. The 2 models agreed that unemployment, long commute, income inequality, number of hospitals, and proportion of natural land were not significantly associated with breast cancer mortality at the county level, with MGWR results not significant for 100% of counties. The methodology was also applied using unadjusted breast cancer mortality rates (2015-2019) as an outcome for comparison. The findings are consistent across both adjusted and unadjusted breast cancer mortality rates (eMethods in Supplement 1).

## Discussion

To our knowledge, this is the first study applying an MGWR model to assess how associations between breast cancer mortality and county-level social determinants vary across space and scale in the US. Based on the SEER age-adjusted rates collected between 2015 and 2019, breast cancer–associated mortality rates differed considerably across the US (Figure 1A and B). Alabama is a clear example of the diverse outcomes experienced by breast cancer patients based on their geographic location even under unified state programs. While the northern part of the state showed significant variation in age-adjusted mortality rates between counties, the southern part of the state displayed more homogeneous rates.

While the MGWR was better at explaining age-adjusted breast cancer mortality in general, both models showed a significant negative and spatially stationary association between breast cancer mortality and access to mammogram screening. Similarly, county-level obesity emerged as a variable with a positive association with breast cancer mortality that had a stationary effect across the US, but that the association had slightly higher effect sizes in the Southern states. Association between obesity and breast cancer incidence and mortality have been thoroughly examined in epidemiological, clinical, and preclinical studies.^26,27,28,29^

Of interest, lifestyle factors that affect obesity, like the food environment index and exercise opportunities were also negatively associated with breast cancer mortality in the OLS and MGWR models. However, their effects were spatially nonstationary with regional-scale variation (Figure 3). For example, food environment index, a variable that combines both physical and financial access to healthy foods, effect sizes for the association with reduced mortality were especially pronounced in areas that have previously been reported as cancer hot spots for non-Hispanic Black women,^30^ such as areas along the Mississippi river, rural southern Virginia, and North Carolina (Figure 1B). Thus, our results indicate that more comprehensive and geographically targeted public health programs with a combined approach that seeks to both increase access to healthy and nutritional foods in underserved areas^31^ and modify eating habits^32,33,34^ could support filling the cancer disparity gap in this region. This highlights the importance of considering spatial nonstationarity of cancer mortality rates.

Access to physical exercise opportunities also emerged as a nonstationary risk factor associated with breast cancer mortality (Figure 3). The beneficial effect of exercise and physical activity have been thoroughly described in the context of breast cancer incidence and mortality, including in individuals harboring genomic alterations of the *BRCA1* and *BRCA2* genes.^35,36,37,38,39,40^ Meta-analyses have provided suggestive evidence that links availability of and engagement in physical activity with improved outcome for breast cancer patients.^41,42,43^ Our MGWR model results indicated that access to exercise opportunities has a positive impact on breast cancer survivorship in areas highly populated by Latino and indigenous Native American communities, like New Mexico, Texas, and Florida, and at the 4 corners between New Mexico, Colorado, and Arizona. Understanding the effects of physical activity on breast cancer mortality in women of different ethnic background may open new opportunities for developing culturally specific educational programs.^44,45,46,47^

### Strengths and Limitations

While numerous studies have assessed social determinants of breast cancer mortality, most previous analyses were either limited to specific geographic areas or were conducted under the assumption that mortality determinants are spatially stationary. Our analysis provides unique insights on the spatial and scale-dependent relationship between health determinants and breast cancer mortality.

Because breast cancer death rates are relatively rare events in the general population, a few limitations of this study need to be addressed. While the SEER database remains the most reliable and comprehensive source of cancer-related mortality data across the US, to protect patients’ confidentiality, mortality rates are not reported for less populated areas where death totals do not reach the minimum reporting threshold. While we tested several approaches for imputing missing data, we found that imputation risked inflating mortality rates in counties with small populations or decreased the spatial variance that is observed in the nonimputed data. Thus, our analysis is biased toward counties that have 10 or more deaths in 5 years and may affect our findings.

In addition, most variables included in our analysis were measured at the county level, not specifically in women at risk for or affected by breast cancer, which may have affected our estimates. We also note that our final MGWR produces a moderate coefficient of determination, especially using the age-adjusted mortality rates as a dependent variable. This is likely due to the complexity of breast cancer mortality and determinants, producing variation that is difficult to capture in models. This is reflected in similar studies that use county-level data that also report moderate model performance,^10,11^ but in general, especially when using individual level data, studies often choose not to report it at all.

Even with these limitations, the MGWR model demonstrated that factors known to be associated with breast cancer have heterogenous effects across geographic regions. By accounting for the inherent spatial distribution of risk factors, population diversity, and their effect on mortality, the MGWR model provides unique opportunities for identifying trends and conceiving policies and health interventions that target specific population characteristics.

## Conclusions

In this cross-sectional study, we found county-level age-adjusted breast cancer mortality rates were significantly positively associated with obesity and negatively associated with proportion of adults screened via mammograms, and that this association was spatially stationary. Smoking, food environment index, exercise opportunities, segregation, mental health care physician ratio, and primary care physician ratio were negatively associated with breast cancer mortality, and light pollution was positively associated. However, the MGWR revealed that the magnitude of effect and significance of these variables varied across geographical regions.

Devising new approaches to address health disparities is a growing priority in cancer research. It is well known that health disparities are driven by complex and often interrelated factors. Untangling these complex relationships requires innovative and multidisciplinary approaches able to tie place-specific factors with disease-related outcomes. The MGWR approach proposed brought a novel perspective for capturing the spatial interrelations between individuals and contextual factors on a large geographic scale. As suggested by our analysis, this approach may have an unparalleled ability to identify vulnerable populations and geographic areas where targeted interventions may lead to healthier communities.

## Notes

Supplement 1. **eAppendix.** Data Processing and Aggregation **eTable 1.** Variables, Description, Source, and Year **eFigure 1.** Variable Selection Process
eMethods.
**eTable 2.** OLS Results Using Crude Female Breast Cancer Mortality as the Dependent Variable **eTable 3.** MGWR Results Using Crude Female Breast Cancer Mortality as the Dependent Variable **eFigure 2.** MGWR Standardized Beta Coefficients Describing the Association Between Crude Breast Cancer Mortality (Person Years at Risk) and Obesity, Food Index, Mammogram Testing, and Female Population Over Age 65 **eFigure 3.** MGWR Standardized Beta Coefficients Describing the Association Between Crude Breast Cancer Mortality (Person Years at Risk) and Exercise, Primary Care Physicians’ Ratio, Unemployment, and Income Inequality
eReferences.

Supplement 2.
Data Sharing Statement
