# Supplementary material for: Geographical Variation in Social Determinants of Female Breast Cancer Mortality Across US Counties
Source: JAMA Netw Open. 2023 Sep 14;6(9):e2333618. doi: 10.1001/jamanetworkopen.2023.33618 (PMC10502521; doi:10.1001/jamanetworkopen.2023.33618)
Supplement: Supplement 1. — eAppendix. Data Processing and Aggregation eTable 1. Variables, Description, Source, and Year eFigure 1. Variable Selection Process eMethods. eTable 2. OLS Results Using Crude Female Breast Cancer Mortality as the Dependent Variable eTable 3. MGWR Results Using Crude Female Breast Cancer Mortality as the Dependent Variable eFigure 2. MGWR Standardized Beta Coefficients Describing the Association Between Crude Breast Cancer Mortality (Person Years at Risk) and Obesity, Food Index, Mammogram Testing, and Female Population Over Age 65 eFigure 3. MGWR Standardized Beta Coefficients Describing the Association Between Crude Breast Cancer Mortality (Person Years at Risk) and Exercise, Primary Care Physicians’ Ratio, Unemployment, and Income Inequality eReferences. [file jamanetwopen-e2333618-s001.pdf]

# Supplemental Online Content

Anderson T, Herrera D, Mireku F, et al. Geographical variation in social determinants of female breast cancer mortality across US counties. *JAMA Netw Open.* 2023;6(9):e2333618. doi:10.1001/jamanetworkopen.2023.33618

**eAppendix.** Data Processing and Aggregation

**eTable 1.** Variables, Description, Source, and Year

**eFigure 1.** Variable Selection Process

**eMethods.**

**eTable 2.** OLS Results Using Crude Female Breast Cancer Mortality as the Dependent Variable

**eTable 3.** MGWR Results Using Crude Female Breast Cancer Mortality as the Dependent Variable

**eFigure 2.** MGWR Standardized Beta Coefficients Describing the Association Between Crude Breast Cancer Mortality (Person Years at Risk) and Obesity, Food Index, Mammogram Testing, and Female Population Over Age 65

**eFigure 3.** MGWR Standardized Beta Coefficients Describing the Association Between Crude Breast Cancer Mortality (Person Years at Risk) and Exercise, Primary Care Physicians' Ratio, Unemployment, and Income Inequality

**eReferences.**

This supplemental material has been provided by the authors to give readers additional information about their work.

eAppendix. Data Processing and Aggregation

Data for each of the 56 county level variables is described in eTable 1. For each variable, all available datasets from 2000-2019 were collected, cleaned and aggregated using various software including R[1], PostgreSQL[2], and ArcGIS Pro[3]. It should be noted, that due to publication lag in datasets, the datasets that are published in one year may capture a previous year or set of years. For example, the 2018 SVI dataset is really an average of several datasets from 2014 to 2018. In the case that multiple datasets over time for a single variable were collected, the values for each county across all datasets for the same variable are averaged. The flow of the variables through to the final regression models is captured in eFigure 1.

© 2023 Anderson T et al. *JAMA Network Open.*

**eTable 1.** Variables, description, source, and year

| Variable | Source | Description | Date |
| --- | --- | --- | --- |
| **1. %Uninsured** | **SVI[4] (EP_UNINSUR)** | **Uninsured in the total civilian noninsured in the total civilian noninstitutionalized population estimates 2014-2018** | **2018 dataset (2014-2018)** |
| 2. %No High School Diploma | SVI (EP_NOHSDDP) | Percentage Age 25+ with no high school diploma estimate (2014-2018) | 2018 dataset (2014-2018) |
| 3. %Minority | SVI (EP_MINRTY) | Percent except white, non-Hispanic white | 2018 dataset (2014-2018) |
| **4. %Disability** | **SVI (EP_DISABL)** | **Percent Civilian Noninstitutionalized population with a disability estimate (2014-2018)** | **2018 dataset (2014-2018)** |
| **5. %Adult Smoking** | **CHR[5]** | **Percentage of adults that reported currently smoking** | **2019 dataset (from 2016)<br>2017 dataset (from 2015)<br>2016 dataset (from 2014)** |
| 6. %Excessive drinking | CHR | Percentage of adults that report excessive drinking | 2019 dataset (from 2016)<br>2017 dataset (from 2015)<br>2016 dataset (from 2014) |
| 7. Poor mental health days | CHR | Average number of reported mentally unhealthy days per month | 2019 dataset (from 2016)<br>2017 dataset (from 2015)<br>2016 dataset (from 2014) |
| **8. %Mammography Screening** | **CHR** | **Percentage of adults screened (that have Medicare/by race/ethnicity)** | **2019 data (from 2016)<br>2018 data (from 2014)<br>2016 data (from 2013)<br>2015 data (from 2012)<br>2014 data (from 2011)<br>2013 data (from 2010)** |
| **9. %Adult Obesity** | **CHR** | **Percentage of adults that report BMI >= 30** | **2019 data (from 2015)<br>2018 data (from 2014)<br>2017 data (from 2013)<br>2016 data (from 2012)<br>2015 data (from 2011)<br>2014 data (from 2010)** |
| 10. Violent crime | CHR | Number of violent crimes | 2019 data (from 2014 & 2016)<br>2016 data (from 2010-2012) |
| **11. %Long commute – driving alone** | **CHR** | **Among workers who commute in their car alone, the percentage that commute more than 30 minutes** | **2019 data (from 2013-2017)** |
| 12. %Severe Housing Problems | CHR | Percentage of households with at least 1 of 4 housing problems: overcrowding, high housing costs, lack of kitchen facilities, or lack of plumbing facilities. | 2019 data (from 2011-2015) |

| | | | |
|---|---|---|---|
| **13. Food environment index** | **CHR** | **Index of factors that contribute to a healthy food environment, from 0 (worst) to 10 (best)** | **2019 data (from 2015 & 2016)**<br>**2017 data (from 2014)**<br>**2016 data (from 2013)**<br>**2015 data (from 2012)**<br>**2014 data (from 2010-2011)** |
| **14. %Access to exercise opportunities** | **CHR** | **Percentage of population with adequate access to locations for physical activity.** | **2019 data (from 2010 & 2018)** |
| **15. Primary care physicians** | **CHR** | **Population to physician ratio** | **2019 data (from 2016)**<br>**2018 data (from 2015)**<br>**2017 data (from 2014)**<br>**2016 data (from 2013)**<br>**2015 data (from 2012)**<br>**2014 data (from 2011)**<br>**2013 data (from 2010)** |
| **16. Mental health Providers** | **CHR** | **Population to mental health providers ratio** | **2019 data (from 2018)**<br>**2018 data (from 2017)**<br>**2017 data (from 2016)**<br>**2016 data (from 2015)**<br>**2015 data (from 2014)** |
| 17. %Education (Some College) | CHR | Percentage of adults age 25-44 with some post-secondary education | 2019 data (from 2013-2017) |
| 18. National Cancer Center, count | gis.cancer.gov[6] | Count of NCIs in each county | 2022 |
| 19. Clinical trials | Clinicaltrials.gov[7] | Number of clinical trials per county over the past 10 years; Number of clinical trials is set to 0 if <10 (less than 1 per year) | 2010-2019 |
| **20. Income Inequality** | **CHR** | **Ratio of household income at the 80$^{th}$ percentile to income at the 20$^{th}$ percentile** | **2019 data (from 2013-2017)** |
| 21. Race (% White) | ACS[8] (DP05_0032PE) | Percentage one race (level) | 2020 5YE (2015-2019)<br>2015 5YE (2010-2014) |
| 22. Race (% Black) | ACS (DP05_0033PE) | Percentage one race (level) | 2020 5YE (2015-2019)<br>2015 5YE (2010-2014) |
| 23. Race (% Indigenous) | ACS (DP05_0034PE) | Percentage one race (level) | 2020 5YE (2015-2019)<br>2015 5YE (2010-2014) |
| 24. Race (% Asian) | ACS (DP05_0039PE) | Percentage one race (level) | 2020 5YE (2015-2019)<br>2015 5YE (2010-2014) |

© 2023 Anderson T et al. *JAMA Network Open.*

| | | | |
|---|---|---|---|
| 25. Race (% Islander) | ACS (DP05_0047PE) | Percentage one race (level) | 2020 5YE (2015-2019)<br>2015 5YE (2010-2014) |
| 26. Race (% Other) | ACS (DP05_0052PE) | Percentage one race (level) | 2020 5YE (2015-2019)<br>2015 5YE (2010-2014) |
| 27. Marital Status (% Now married) | ACS (S1201_C02_001E) | Estimates ("%") of Marital status of 15 years and above. | 2020 5YE (2015-2019)<br>2015 5YE (2010-2014) |
| 28. Marital Status (% Widowed) | ACS (S1201_C03_001E) | Estimates ("%") of Marital status of 15 years and above. | 2020 5YE (2015-2019)<br>2015 5YE (2010-2014) |
| 29. Marital Status (% Divorced) | ACS(S1201_C04_001E) | Estimates ("%") of Marital status of 15 years and above. | 2020 5YE (2015-2019)<br>2015 5YE (2010-2014) |
| 30. Marital Status (% Never married) | ACS (S1201_C06_001E) | Estimates ("%") of Marital status of 15 years and above. | 2020 5YE (2015-2019)<br>2015 5YE (2010-2014) |
| 31. Marital Status (%<br>32. Separated) | ACS (S1201_C05_001E) | Estimates ("%") of Marital status of 15 years and above. | 2020 5YE (2015-2019)<br>2015 5YE (2010-2014) |
| **33. Age (% Female age 65+)** | **ACS** | **Estimates of % Female Age 65+** | **2020 5YE (2015-2019)**<br>**2015 5YE (2010-2014)** |
| 34. Premature age adjusted mortality | CHR | Number of deaths among residents under age 75 per 100,000 population (age-adjusted). | 2019 data (from 2015-2017) |
| 35. Social Associations | CHR | Number of membership associations per 100,000 | 2019 data (from 2016)<br>2018 data (from 2015)<br>2017 data (from 2014)<br>2016 data (from 2013)<br>2015 data (from 2012) |
| 36. Segregation Black/White | CHR | Index of dissimilarity where higher values indicated greater residential segregation between black and white county residents | 2019 data (from 2013-2017) |
| **37. Segregation non-white/white** | **CHR** | **Index of dissimilarity where higher values indicated greater residential segregation between non-white and white county residents** | **2019 data (from 2013-2017)** |
| **38. Unemployment** | **CHR** | **Percentage of people ages 16+ unemployed and looking for work,** | **2019 data (from 2017)**<br>**2018 data (from 2016)**<br>**2017 data (from 2015)**<br>**2016 data (from 2014)**<br>**2015 data (from 2013)**<br>**2014 data (from 2012)**<br>**2013 data (from 2011)** |
| 39. Open Water | NLCD[9] | The proportion of each county that was categorized as open water landcover, averaged across years. | 2011, 2013, 2016, 2019 |
| 40. Greenspace | NLCD | The proportion of each county that was categorized as Developed Open Space, Barren Land, Deciduous Forest, Evergreen Forest, Mixed Forest, Dwarf Scrub, Shrub/Scrub, | 2011, 2013, 2016, 2019 |

© 2023 Anderson T et al. *JAMA Network Open.*

| | | | |
|---|---|---|---|
| | | Grassland/Herbaceous, Sedge/Herbaceous, Lichen, Moss, Woody Wetlands, or Emergent Herbaceous Wetlands landcover, averaged across years. | |
| **41. Natural Lands** | **NLCD** | **The sum of the proportion of open water and proportion of open greenspace.** | **2011, 2013, 2016, 2019** |
| 42. Impervious Surface | NLCD | Mean percent impervious cover, averaged across years | 2011, 2013, 2016, 2019 |
| **43. Radiance** | **VIIRS/DNB**[10] | **Mean temporal radiance using observations from snow free periods (nWatts•cm$^{-2}$•sr$^{-1}$), averaged across years** | 2013-2019 |
| 44. Toxic release (lead) | EPA Toxic Release Inventory Program (TRI)[11] | Average annual release of lead substance [Lead (7439-92-1), Lead compounds (N420), Lead and lead compounds (N420), Lead (7439-92-1)] in kg by county | 2011-2019 |
| 45. Toxic release (arsenic) | EPA Toxic Release Inventory Program (TRI) | Average annual release of arsenic substance [Arsenic compounds (N020), Arsenic (7440-38-2), Arsenic and arsenic compounds (N020)] in kg by county | 2011-2019 |
| 46. Toxic release (organic solvents) | EPA Toxic Release Inventory Program (TRI) | Average annual release of organic solvents [Methanol (67-56-1), Acetonitrile (75-05-8), 2-Methoxyethanol (109-86-4), 2-Ethoxyethanol (110-80-5), Methanol (67-56-1), Formaldehyde (50-00-0), Certain glycol ethers (N230), Methyl tert-butyl ether (1634-04-4), Bis(2-chloroethyl) ether (111-44-4), 4,4'-Diaminodiphenyl ether (101-80-4), Chloromethyl methyl ether (107-30-2), Bis(2-chloro-1-methylethyl) ether (108-60-1), Diglycidyl resorcinol ether (101-90-6), Bis(chloromethyl) ether (542-88-1), Chloroform (67-66-3), Ethyl chloroformate (541-41-3), Bromoform (75-25-2)] in kg by county | 2011-2019 |
| 47. Toxic release (zinc) | EPA Toxic Release Inventory Program (TRI) | Average annual release of zinc substance [Zinc compounds (N982), Zinc (fume or dust) (7440-66-6), Zinc and zinc compounds (N982)] in kg by county | 2011-2019 |
| **48. Transit Stops** | **OpenStreetMap**[12] | **Total bus stations, bus stops, railway stations, and tram stops by county.** | **2022** |
| 49. Paths | OpenStreetMap | Total length (km) of pedestrian/bike paths (path, cycleway, pedestrian, footway). | 2022 |
| **50. Grocery Stores** | **SafeGraph**[13] | **Number of grocery stores** | **2021** |
| **51. Hospitals** | **SafeGraph** | **Number of hospitals** | **2021** |
| 52. Community Centers | SafeGraph | Number of community centers | 2021 |

© 2023 Anderson T et al. *JAMA Network Open.*

| | | | |
|---|---|---|---|
| 53. Metal Manufacturing | SafeGraph | Number of metal manufacturing facilities | 2021 |
| 54. Textile Manufacturing | SafeGraph | Number of textile manufacturing facilities | 2021 |
| 55. Plastic Manufacturing | SafeGraph | Number of plastic manufacturing facilities | 2021 |
| 56. Fast Food | SafeGraph | Number of fast food eateries | 2021 |
| 57. Worship | SafeGraph | Number of places of worship | 2021 |

Final variables used in the models are in bold.

© 2023 Anderson T et al. *JAMA Network Open.*

**eFigure 1.** Variable Selection Process

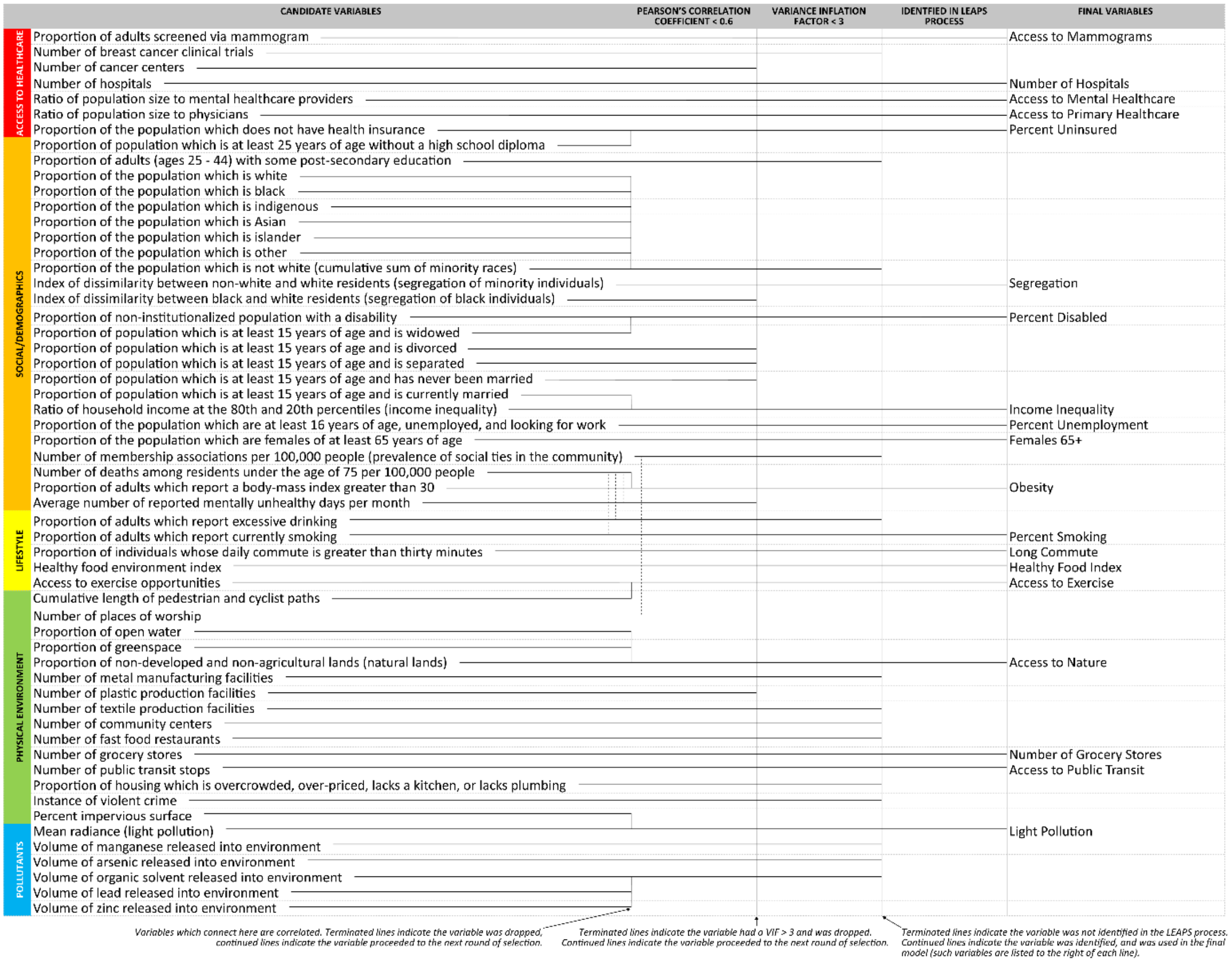


© 2023 Anderson T et al. *JAMA Network Open.*

**eMethods.**

**1.1. Moran's I Cluster and Outlier Analysis**

Local Moran's I statistic, also known as Local Indicators of Spatial Association (LISA) identifies clusters of features that are alike i.e. counties with low breast cancer mortality surrounded by other counties with low breast cancer mortality and are dissimilar i.e. counties with low breast cancer mortality surrounded by other counties with high breast cancer mortality. Clusters are referred to as features that are positively spatially autocorrelated where outliers are negatively spatially autocorrelated. Formally, the Local Moran's I statistic[14]is as follows:

$$I_i = \frac{x_i - \bar{X}}{S_i^2} \sum_{j=1, j\neq i}^{n} w_{i,j}(x_i - \bar{X})$$

where $x_i$is an attribute for feature *i*, $\bar{X}$is the mean of the corresponding attribute, $w_{i,j}$ is the spatial weight between feature i and j. The Local Moran's I was computed using ArcGIS Pro. V. 3.1.0[15].

**1.2. OLS**

Independent variables that do not have a normal distribution are log transformed. Additionally, independent variables are standardized to better compare between coefficients. We use Ordinary Least Squares (OLS) multi-linear regression model to as a baseline for which to compare the MGWR model. OLS is expressed as:

$$y_i = \beta_0 + \beta_{x_i} + \varepsilon_i$$

where $y_i$ is the number of deaths per 100,000 women per year at county *i;* $\beta_0$ is the intercept, β is the estimated rate of change between explanatory variable *x* and *y* for county *i* and $\varepsilon_i$ is the error term. We examined the resulting model for multicollinearity across all independent variables so that variance inflation factor (VIF) < 3. The OLS model was computed using ArcGIS Pro. v. 3.1.0[16].

**1.3. MGWR**

MGWR[17] computes a local regression model for every county *i* in the dataset by borrowing data from other surrounding counties *j* that fall within county *i*'s neighborhood. The neighborhood from which data will be borrowed is defined using a bi-square kernel with a bandwidth i.e., the number of nearest neighbors *j*. The bandwidth was optimized using the golden-section search algorithm to find the number of nearest neighbors that minimize the corrected Akaike Information Criterion (AICc).

Formally, the MGWR model is expressed as:

$$y_i = \sum_{j=1}^{M} \beta_{bwj} X_{ij} + \varepsilon_i$$

where $\beta_{bwj}$ is the estimation of the coefficient for county *i* and *bwj* is the *j*th optimal bandwidth. The resulting bandwidths provide important information on the scale at which certain processes occur. Smaller bandwidths indicate more local processes and larger indicate more global. This information can help to assess the scale at which health interventions may be better addressed. The MGWR model was computed using ArcGIS Pro v. 3.1.0[18].

**2. Results Using Crude Mortality Rate**

Using the crude female breast cancer mortality rates as the dependent variable, the linear model ($R^2$ = 0.38, adjusted $R^2$ = 0.38, AICc = 14607.734) results are presented in Table 1 and the MGWR model ($R^2$ = 0.52, adjusted $R^2$ = 0.48, AICc = 14373.063) results are presented in Table 2. Both models outperform the models that use age adjusted female breast cancer mortality rates. In the MGWR, the variables in which the coefficients for 100% of counties are significant include obesity (eFigure 2A), food index (eFigure 2B), mammograms (eFigure 2C), and female

© 2023 Anderson T et al. *JAMA Network Open.*

population over the age of 65 (eFigure 2D). The MGWR algorithm uses very large bandwidths for these variables, meaning that the relationship between these variables and breast cancer mortality are consistent across the entire US and are thus spatially stationary. In both the OLS and MGWR models, there is a strong positive and statistically significant association between obesity and female population over the age of 65 and breast cancer mortality, meaning that as the independent variable increases, breast cancer mortality also increases. Additionally, in both models there is a strong negative and statistically significant association between food index and the number of mammogram tests and breast cancer mortality, meaning that as the independent variable increases, breast cancer mortality decreases.

The OLS and MGWR model using the crude mortality rates as a dependent variable agree that *in general* there is a negative association between the independent variables smoking, exercise opportunities, segregation, mental health care physician ratio, and primary care physician ratio and breast cancer mortality. The OLS and MGWR models also agree that in general there is a positive association between number of grocery stores and breast cancer mortality. Yet, the MGWR reveals that for some counties the coefficients for these six variables are not significant. Furthermore, the MGWR reveals that the trends and magnitude of these variable's coefficients vary from county to county. Thus, the relationship between these variables and breast cancer mortality can be considered as non-spatially stationary and as predictors are place dependent. The MGWR provides insights into the locations where each variable makes a significant strong positive or negative impact on breast cancer mortality. For example, for most of the US, the association between access to exercise and breast cancer mortality is not significant, but some locations in the Great Planes, the South, and most of Florida exhibit a strong negative association (eFigure 3A). In another example, while primary care physician ratio and breast cancer mortality is not significant for most of the US, a strong negative association can be observed in the Mid-Atlantic and Northeastern US (eFigure 3B).

The two models using crude mortality rates agree that long commute, number of hospitals, and proportion of natural land is not a significant predictor of breast cancer mortality at the county level. Where OLS finds unemployment and disability not significant, the MGWR finds that these variables are significant in some locations and on average is positively associated with breast cancer mortality. For example, there is a strong positive association between unemployment and breast cancer mortality in Alabama, Mississippi, Louisiana, and parts of Florida, which may suggest reliance on employment benefits for care in these locations. However, due to non-spatial stationarity, we also observe a strong negative association between unemployment and breast cancer mortality in the western US (eFigure 3C). Likewise, where OLS finds income inequality is not significant, the MGWR finds that this variable is significantly negatively associated with breast cancer mortality in the Mid-Atlantic and Northeastern US such as Pennsylvania, New York, Vermont as well as parts of Utah, suggesting strong health programs for low-income individuals in these locations (eFigure 3D). Finally, where OLS finds a positive association between mean radiance and breast cancer mortality and a negative association between the number of transit stops and breast cancer mortality, the MGWR does not find the coefficients for these variables statistically significant for any county in the US.

© 2023 Anderson T et al. *JAMA Network Open.*

*eTable 2. OLS results using crude female breast cancer mortality as the dependent variable.*

| **Variable Name** | **est.** | **p-value** | **st. error** | **VIF** |
|---|---|---|---|---|
| intercept | 32.667 | 0.000 | 0.225 | ~ |
| **Lifestyle** | | | | |
| smoking, % adults | -0.915 | 0.000 | 0.241 | 1.977 |
| obesity, % adults | 1.655 | 0.000 | 0.240 | 2.680 |
| food environment index, 0 = worst, 10 = best | -1.372 | 0.000 | 0.272 | 2.695 |
| long commute, % workers | -0.049 | 0.796 | 0.188 | 1.544 |
| exercise opportunities, % pop | -0.793 | 0.002 | 0.258 | 2.254 |
| **Demographic** | | | | |
| unemployment, % 16+ | -0.340 | 0.130 | 0.224 | 1.835 |
| segregation, all/White, higher = greater | -0.982 | 0.000 | 0.208 | 1.223 |
| disability, % pop | 0.301 | 0.270 | 0.272 | 3.000 |
| female > age 65 | 4.890 | 0.000 | 0.222 | 1.930 |
| income inequality (80th percentile), ratio to 20th | -0.340 | 0.097 | 0.205 | 1.838 |
| **Access to Health Care** | | | | |
| uninsured, % pop | -0.830 | 0.000 | 0.211 | 1.600 |
| mammograms, % adults screened | -2.165 | 0.000 | 0.329 | 1.610 |
| mental health care physicians, ratio to pop | -1.500 | 0.000 | 0.364 | 1.405 |
| primary care physicians, ratio to pop | -1.522 | 0.001 | 0.478 | 1.570 |
| number of hospitals, per capita | -0.162 | 0.454 | 0.216 | 1.140 |
| **Environment** | | | | |
| mean radiance, $nWatts \cdot cm^{-2} \cdot sr^{-1}$ | 0.551 | 0.001 | 0.165 | 1.684 |
| no. transit stops, per capita | -0.608 | 0.001 | 0.186 | 1.845 |
| proportion of natural land per county | -0.198 | 0.300 | 0.191 | 1.540 |
| no. grocery stores, per capita | 0.857 | 0.002 | 0.271 | 1.214 |

© 2023 Anderson T et al. *JAMA Network Open.*

*eTable 3. MGWR results using crude female breast cancer mortality as the dependent variable.*

| Variable Name | sig. counties (%) | bandwidth size | mean est. | st. dev. est. | min est. | max est. |
|---|---|---|---|---|---|---|
| intercept | 100.000 | 85.000 | 32.453 | 1.538 | 27.534 | 38.545 |
| **Lifestyle** | | | | | | |
| smoking, % adults | 22.385 | 298.000 | -1.146 | 1.122 | -3.301 | 1.778 |
| obesity, % adults | 100.000 | 2180.000 | 0.711 | 0.014 | 0.673 | 0.743 |
| food environment index, 0 = worst, 10 = best | 100.000 | 2180.000 | -1.544 | 0.045 | -1.582 | -1.416 |
| long commute, % workers | 0.000 | 2180.000 | 0.140 | 0.019 | 0.074 | 0.180 |
| exercise opportunities, % pop | 12.477 | 252.000 | -0.772 | 1.117 | -4.487 | 1.613 |
| **Demographic** | | | | | | |
| unemployment, % 16+ | 22.202 | 971.000 | 0.306 | 0.766 | -1.181 | 1.567 |
| segregation, all/White, higher = greater | 28.211 | 731.000 | -0.847 | 0.638 | -2.979 | -0.092 |
| disability, % pop | 63.853 | 1239.000 | 1.079 | 0.598 | 0.009 | 1.799 |
| female > age 65 | 100.000 | 1553.000 | 4.435 | 0.620 | 3.708 | 5.542 |
| income inequality (80th percentile), ratio to 20th | 17.569 | 1553.000 | -0.481 | 0.167 | -0.814 | -0.166 |
| **Access to Health Care** | | | | | | |
| uninsured, % pop | 0.000 | 2180.000 | -0.312 | 0.052 | -0.441 | -0.240 |
| mammograms, % adults screened | 100.000 | 2106.000 | -1.844 | 0.158 | -2.025 | -1.501 |
| mental health care physicians, ratio to pop | 13.486 | 252.000 | -0.622 | 2.005 | -6.145 | 5.469 |
| primary care physicians, ratio to pop | 27.890 | 851.000 | -1.037 | 1.532 | -3.995 | 1.297 |
| number of hospitals, per capita | 0.000 | 1165.000 | 0.073 | 0.296 | -0.399 | 0.706 |
| **Environment** | | | | | | |
| mean radiance, nWatts•cm$^{-2}$•sr$^{-1}$ | 0.000 | 2180.000 | 0.258 | 0.057 | 0.200 | 0.445 |
| no. transit stops, per capita | 0.000 | 2180.000 | -0.366 | 0.051 | -0.391 | -0.181 |
| proportion of natural land per county | 0.000 | 2180.000 | -0.082 | 0.041 | -0.197 | -0.041 |
| no. grocery stores, per capita | 54.037 | 2180.000 | 0.617 | 0.046 | 0.515 | 0.695 |

© 2023 Anderson T et al. *JAMA Network Open.*

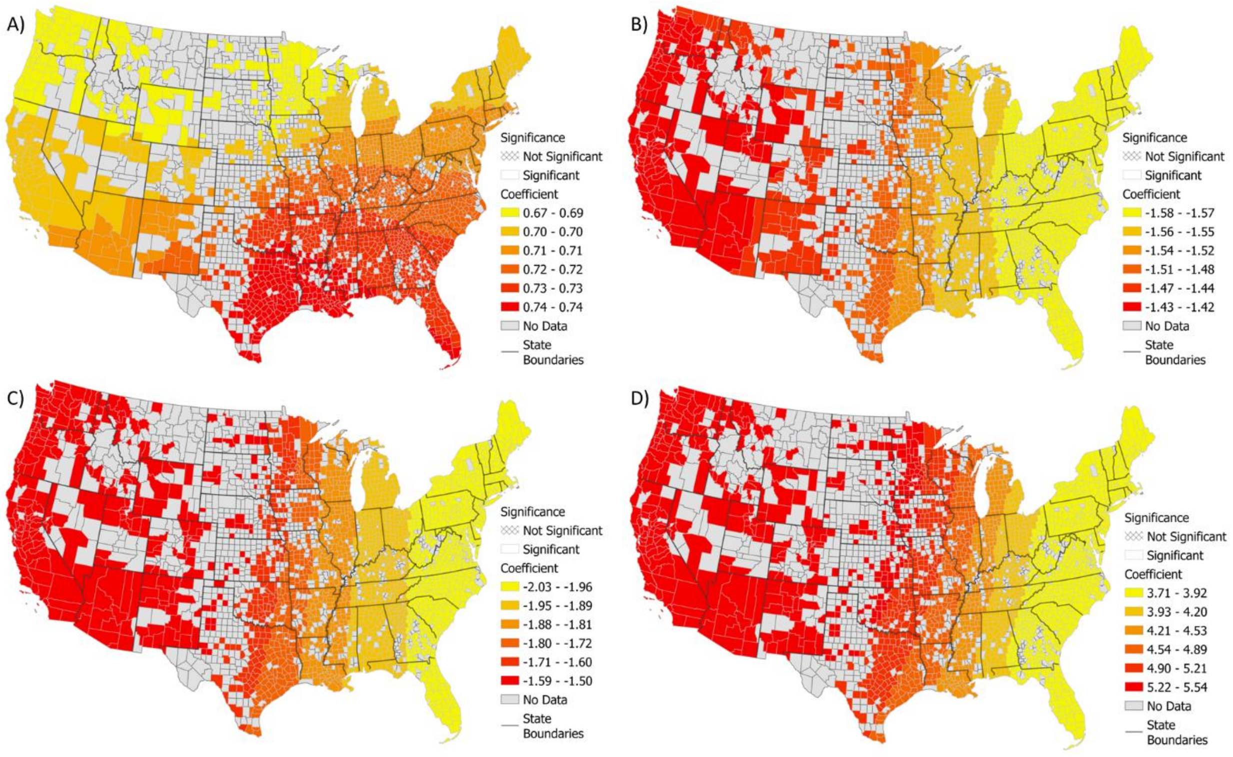


*eFigure 2. MGWR standardized beta coefficients describing the association between crude breast cancer mortality (person years at risk) and A) obesity, B) food index, C) mammogram testing and D) female population over age 65.*

© 2023 Anderson T et al. *JAMA Network Open.*

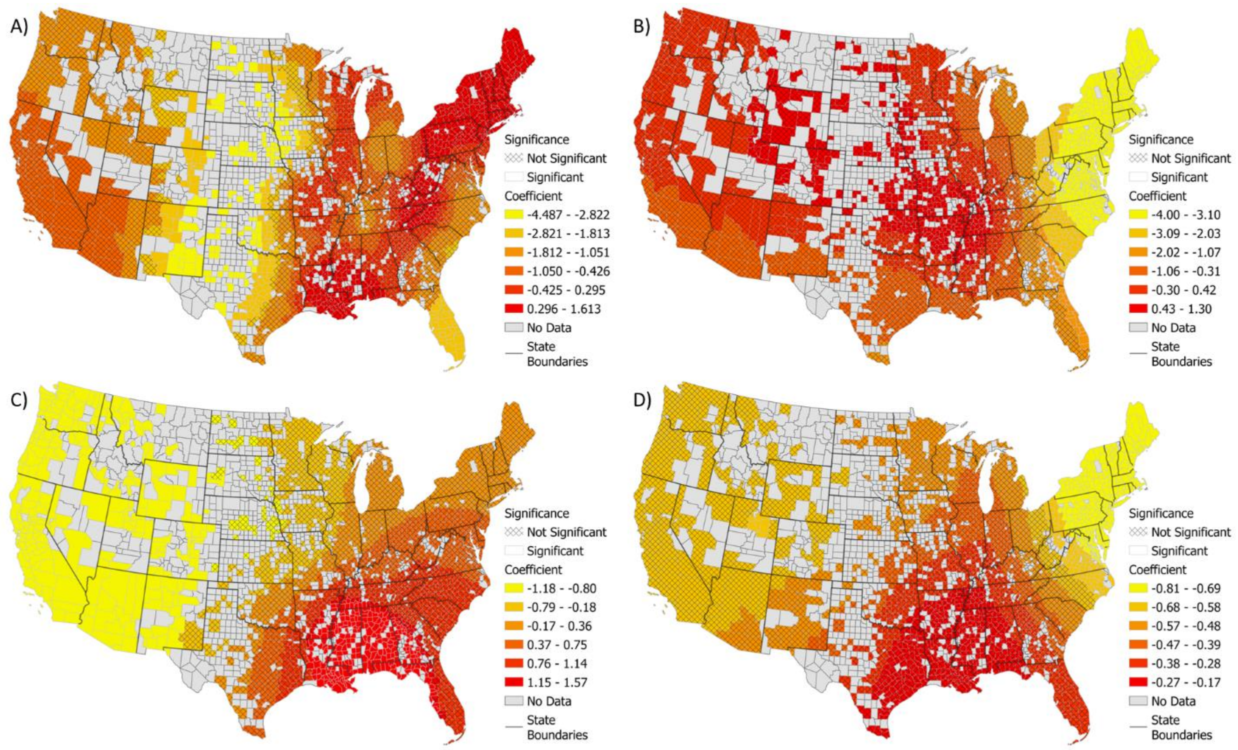


*eFigure 3. MGWR standardized beta coefficients describing the association between crude breast cancer mortality (person years at risk) and A) exercise, B) primary care physicians' ratio, C) unemployment and D) income inequality.*

© 2023 Anderson T et al. *JAMA Network Open.*

**References**


1. *R: A Language and Environment for Statistical Computing* [Computer software]. Version 2.2.1. Vienna, Austria: R Foundation for Statistical Computing; 2022.

2. *PostgreSQL* [Computer software]. Version 14.7. 2022.

3. *ArcGIS Pro* [Computer software]. Version 3.1.0. Redlands, CA: Esri; 2022.

4. Agency for Toxic Substances and Disease Registry. CDC/ATSDR Social Vulnerability Index. Published online 2018. Accessed July 2022. https://www.atsdr.cdc.gov/placeandhealth/svi/index.html

5. University of Wisconsin Population Health Institute. County health rankings & roadmaps. Published online 2019. Accessed July 2022. https://www.countyhealthrankings.org/

6. National Cancer Institute. GIS Portal for Cancer Research. Published online 2022. Accessed July 2022. https://gis.cancer.gov/data

7. U.S. National Library of Medicine. ClinicalTrials.gov. Published online 2019. Accessed July 2022. https://clinicaltrials.gov/

8. United States Census Bureau. American Community Survey. Published online 2019. Accessed July 2022. https://www.census.gov/programs-surveys/acs

9. Multi-Resolution Land Characteristics Consortium. National Land Cover Database. Published online 2019. Accessed July 2022. https://www.mrlc.gov/data

10. NASA Black Marble. VIIRS/NPP Lunar BRDF-Adjusted Nighttime Lights. Published online 2019. Accessed July 2022. https://blackmarble.gsfc.nasa.gov/

11. United States Environmental Protection Agency. Toxic Release Inventory (TRI) Program. Published online 2019. Accessed July 2022. https://www.epa.gov/toxics-release-inventory-tri-program

12. OpenStreetMap contributors. OpenStreetMap United States of America. Published online 2022. Accessed July 2022. https://download.geofabrik.de/north-america/us.html

13. Safegraph. Places POI data. Published online 2021. Accessed July 2022. https://www.safegraph.com/

14. Anselin L. Local Indicators of Spatial Association-LISA. *Geogr Anal*. 1995;27(2):93-115. doi:10.1111/j.1538-4632.1995.tb00338.x

15. Esri. Cluster and Outlier Analysis (Anselin Local Moran's I) (Spatial Statistics)—ArcGIS Pro | Documentation. Published February 2023. Accessed February 2023. https://pro.arcgis.com/en/pro-app/latest/tool-reference/spatial-statistics/cluster-and-outlier-analysis-anselin-local-moran-s.htm

16. Esri. Generalized Linear Regression (Spatial Statistics)—ArcGIS Pro | Documentation. Published February 2023. Accessed February 2023. https://pro.arcgis.com/en/pro-app/latest/tool-reference/spatial-statistics/generalized-linear-regression.htm

17. Fotheringham AS, Yang W, Kang W. Multiscale Geographically Weighted Regression (MGWR). *Ann Am Assoc Geogr*. 2017;107(6):1247-1265. doi:10.1080/24694452.2017.1352480

18. Esri. Multiscale Geographically Weighted Regression (MGWR) (Spatial Statistics)—ArcGIS Pro | Documentation. Published February 2023. Accessed February 2023. https://pro.arcgis.com/en/pro-app/latest/tool-reference/spatial-statistics/multiscale-geographically-weighted-regression.htm


© 2023 Anderson T et al. *JAMA Network Open.*
