# Supplementary material for: Geographical Variation in Social Determinants of Female Breast Cancer Mortality Across US Counties
Source: JAMA Netw Open. 2023 Sep 14;6(9):e2333618. doi: 10.1001/jamanetworkopen.2023.33618 (PMC10502521; doi:10.1001/jamanetworkopen.2023.33618)
Supplement: Supplement 2. — Data Sharing Statement [file jamanetwopen-e2333618-s002.pdf]

Data Sharing Statement

Anderson. Geographical Variation in Social Determinants of Female Breast Cancer Mortality Across US Counties. *JAMA Netw Open*. Published September 14, 2023. doi:10.1001/jamanetworkopen.2023.33618

Data
Data available: No

Additional Information
Explanation for why data not available: The data is open and available for public use.
